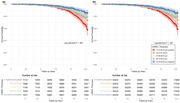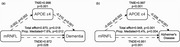# Thinning of the Retinal Nerve Fiber Layer and Incident Dementia Over 12 Years: A Prospective Cohort Study of 35 433 Participants

**DOI:** 10.1002/alz70856_097474

**Published:** 2025-12-24

**Authors:** Qianhua Zhao, Yuzhuo Wang, Xiaoxi Ma, Haoze Cen, Jie Wang, Jie Wu, Ding Ding, Yiqin Xiao

**Affiliations:** ^1^ Huashan Hospital, Fudan University, Shanghai, Shanghai, China; ^2^ National Center for Neurological Disorders, Huashan Hospital, Fudan University, Shanghai, China; ^3^ MOE Frontiers Center for Brain Science, Fudan University, Shanghai, China; ^4^ Institute of Neurology, Huashan Hospital, Fudan University, Shanghai, China; ^5^ National Clinical Research Center for Aging and Medicine, Huashan Hospital, Shanghai, China; ^6^ Institute and department of Neurology, Huashan Hospital, Fudan University, Shanghai, China; ^7^ National Clinical Research Center for Aging and Medicine, Huashan Hospital, Fudan University, Shanghai, China; ^8^ School of Statistics and Data Science, Shanghai University of Finance and Economics, Shanghai, China; ^9^ Institute and department of Neurology, Huashan Hospital, Fudan University, shanghai, China; ^10^ Department of Ophthalmology, Huashan Hospital, Fudan University, Shanghai, Shanghai, China

## Abstract

**Background:**

Thinning of the retinal nerve fiber layer (RNFL) has been linked to cognitive decline, but its relationship with long‐term dementia risk and the potential role of apolipoprotein E (APOE) genotype remain unclear.

**Method:**

This retrospective cohort study analyzed data from the UK Biobank (2006–2010) with follow‐up till 2022. A total of 35,433 participants with qualified macular RNFL (mRNFL) measurements were included, excluding those with eye diseases, baseline dementia, or missing APOE genotype data. Generalized linear regression model (GLM) and the Jonckheere‐Terpstra (JT) test were used to examine the relationship between APOE ε4 and mRNFL thickness. Cox proportional hazards regression and mediation analysis assessed the associations between mRNFL thickness, dementia risk, and APOE genotype. Inverse probability weighting (IPW) was applied in an additional Cox model to address confounding by APOE ε4 status on RNFL thickness, ensuring a more accurate estimation of the relationship with dementia risk.

**Result:**

Over a median follow‐up of 12.49 years, 392 participants (1.11%) developed dementia. APOE ε4 genotype was significantly associated with baseline mRNFL thickness, showing a stepwise decline across ε4 non‐carriers, heterozygotes, and homozygotes (β = −0.14 [95% CI, −0.23 to −0.05]; *p* = .002). Participants in the lowest mRNFL thickness quintile had a 64% higher risk of developing dementia compared to those in the highest quintile (HR, 1.64 [95% CI, 1.17–2.30]; *p* = .004). Each 5‐μm decrease in mRNFL thickness was associated with a 15% increased risk of dementia (HR, 1.15 [95% CI, 1.02–1.30]; *p* = .020). These results were consistent after applying inverse probability weighting (IPW) adjustment. Similar associations were observed for Alzheimer's disease (AD) with thinner mRNFL thickness showing an increased risk. Mediation analysis revealed that most of the effect of RNFL on dementia and AD was independent of APOE, with only 7.6% (95% CI, 2.6% to 28.6%; *p* = .018) of the effect on dementia and 5.4% (95% CI, 2.0% to 15.3%; *p* < .001) of the effect on AD attributable to APOE.

**Conclusion:**

In this population‐based cohort study, the thinning of RNFL was associated with an increased risk of dementia over 12 years, largely independent of APOE status.